# Multi-Target Drugs: The Trend of Drug Research and Development

**DOI:** 10.1371/journal.pone.0040262

**Published:** 2012-06-29

**Authors:** Jin-Jian Lu, Wei Pan, Yuan-Jia Hu, Yi-Tao Wang

**Affiliations:** State Key Laboratory of Quality Research in Chinese Medicine, Institute of Chinese Medical Sciences, University of Macau, Taipa, Macau SAR, China; The University of Texas M. D. Anderson Cancer Center, United States of America

## Abstract

Summarizing the status of drugs in the market and examining the trend of drug research and development is important in drug discovery. In this study, we compared the drug targets and the market sales of the new molecular entities approved by the U.S. Food and Drug Administration from January 2000 to December 2009. Two networks, namely, the target–target and drug–drug networks, have been set up using the network analysis tools. The multi-target drugs have much more potential, as shown by the network visualization and the market trends. We discussed the possible reasons and proposed the rational strategies for drug research and development in the future.

## Introduction

Despite the considerable progress in the high-throughput screening method, the rational drug design, and the massive drug-development efforts, the number of successful drugs did not significantly increase during the past decade [1]. The strategy for screening single-target and highly specific agents was widely researched for some time [2], [3]. However, this effort has not been very successful, and undeniably, the bottleneck lies in the area of drug research and development [2]. Until now, there are still not fully effective drugs for treating complex diseases, such as cancer, metabolic diseases, cardiovascular diseases, and neurological diseases. Thus, we believe that the strategy or models used for new drug discovery have to be reconsidered.

Recent developments in biological systems and overall clinical experience have revealed that the single-target drugs may not always induce the desired effect to the entire biological system even if they successfully inhibit or activate a specific target [1], [2], [4], one reason is that organisms can affect effectiveness through compensatory ways. The development of diseases, particularly the complex ones, involves several aspects. Thus, scientists have recently proposed the multi-target drug design concept [1], [4], [5]. This manuscript aims to determine the status of drug research and development through network views and market sales in the past decade and confirm whether multi-target drugs are the current trend in drug research and development. We also propose rational strategies for future drug research and development.

## Results

### Drug Targets

The total number of sampled new molecular entities (NMEs) approved by the U.S. Food and Drug Administration (FDA) from January 2000 to December 2009 has reached 223. The average target number of sampled drugs is 2.5, which is higher than the 1.8 reported by Yildirim *et al.* using Drugbank data before March 2006 [6], [7]. This increase may partly indicate the rising targets per drug in the recent years.

### Target–target Network

**Figure 1:**
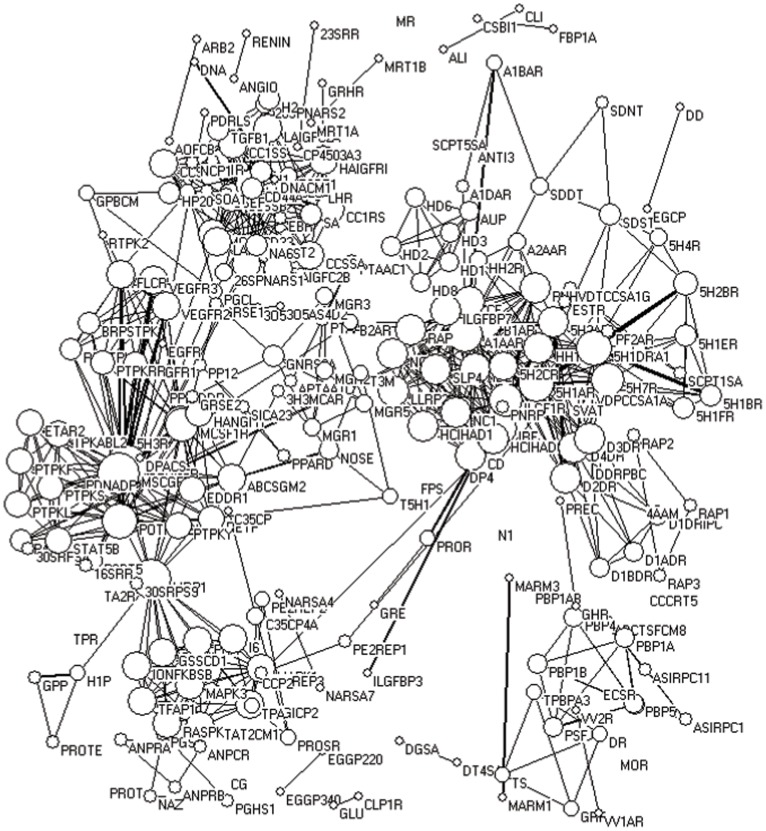
Target–target network. The circles indicate the targets and the size of circles represents nodal degree. The links between the targets represent the number of drugs simultaneously focused by two neighboring targets. Thicker ties mean stronger interactions, whereas thinner links represent weaker relationships.

**Figure 2:**
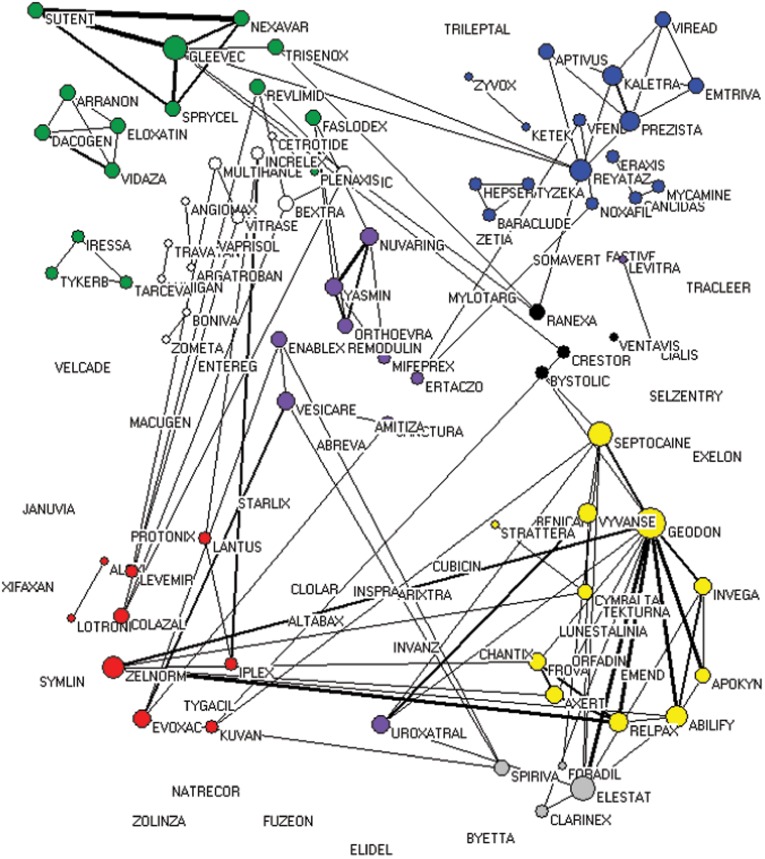
Drug–drug network. The circles indicate the drugs and the size of circles represents nodal degree. The circles of nodes without any line will disappear in the networks because their nodal degree is equal to zero. The links between the drugs represent the number of targets simultaneously focused by the two neighboring drugs. Thicker ties mean stronger interactions, whereas thinner links represent weaker relationships. Red, alimentary tract and metabolism; Yellow, nervous system; Blue, general anti-infectives systemic; Green, antineoplastic and immunomodulating agents; Purple, genito-urinary system and sex hormones; Grey, respiratory system; Black, cardiovascular system; White, others.

The target–target (Figure 1) and drug–drug (Figure 2) networks were built as described in the Materials and Methods section to make a realistic visualization of information and directly determine the connections between targets and drugs, thereby providing important information on the current status of drug discovery.

The targets of the anti-cancer drugs, anti-infection drugs and anti-nervous-system-related -diseases agents, among others, have been effectively separated to some extent (Figure 1). For example, most of the targets for cancer therapy, such as different types of tyrosine kinase, were clustered in the left panel, whereas most of the nervous-system-related targets, such as dopamine receptors, 5-hydroxytryptamine receptor, adrenergic receptors, and histamine receptors, among others, were clustered on the right. The targets for cancer treatment were relatively more scattered than those for other diseases, indicating the complex mechanism involved in cancer development and the diverse methods for cancer chemotherapy.

Most of the targets have connections with the others (at least with one drug) through target–target network visualization, which further confirms the importance of multi-target drugs. Although some drugs were developed based on the single-target strategy, researchers later discovered the diversity of their targets. Their lines were thicker than the others, indicating that more drugs affect these targets. A typical aggregation is that of tyrosin kinases. In fact, several anti-cancer drugs target MCSF1R (macrophage colony-stimulating factor 1 receptor), MSCGFR (mast/stem cell growth factor receptor), POTPKABL1 (proto-oncogene tyrosine-protein kinase ABL1) and VEGFR2 (vascular endothelial growth factor receptor 2), among others. This development exhibits the recent trend of anti-cancer drug discovery. Another remarkable aggregation includes 5H1BR (5-hydroxytryptamine 1B receptor), 5H1DR (5-hydroxytryptamine 1D receptor), 5H2AR (5-hydroxytryptamine 2A receptor), D2DR [D(2) dopamine receptor], D3DR [D(3) dopamine receptor], D4DR [D(4) dopamine receptor], HH1R (histamine H1 receptor), and so on. These receptors are the targets for the treatment of nervous system diseases. These observations also indicate the market demand in the recent years.

We also conducted a centrality analysis and found that ABCSGM2 (ATP-binding cassette sub-family G member 2), NOSE (nitric-oxide synthase, endothelial), P4H (phenylalanine-4-hydroxylase), MRP1 (multi-drug resistance protein 1), A1AAR (Alpha-1A adrenergic receptor), B1AR (Beta-1 adrenergic receptor), among others, present relative high betweenness centrality in the target–target network (Table 1), indicating their importance in this network and the potential of development of new drugs.

**Table 1:** Top 20 betweenness centrality in the target–target network.

Rank	Targets Abbreviation	Targets Full Name	Betweenness Centrality
1	ABCSGM2	ATP-binding cassette sub-family G member 2	0.0395048
2	NOSE	Nitric-oxide synthase, endothelial	0.0390933
3	P4H	Phenylalanine-4-hydroxylase	0.0379617
4	MRP1	Multi-drug resistance protein 1	0.0255478
5	A1AAR	Alpha-1A adrenergic receptor	0.0247959
6	B1AR	Beta-1 adrenergic receptor	0.0131237
7	BPDGFR	Beta platelet-derived growth factor receptor	0.0089909
8	MSCGFR	Beta platelet-derived growth factor receptor	0.0089909
9	5H1AR	5-hydroxytryptamine 1A receptor	0.0047314
10	5H1DR	5-hydroxytryptamine 1D receptor	0.0047314
11	H1P	HIV-1 protease	0.0045266
12	POTPKABL1	Proto-oncogene tyrosine-protein kinase ABL1	0.0041608
13	5H7R	5-hydroxytryptamine 7 receptor	0.0026662
14	APDGFR	Alpha platelet-derived growth factor receptor	0.0021861
15	MCSF1R	Macrophage colony-stimulating factor 1 receptor	0.0021861
16	D4DR	D(4) dopamine receptor	0.0021604
17	D3DR	D(3) dopamine receptor	0.0021604
18	D2DR	D(2) dopamine receptor	0.0021604
19	SDDT	Sodium-dependent dopamine transporter	0.0021565
20	SDST	Sodium-dependent serotonin transporter	0.0006687

### Drug–drug Network

Drugs used in treating the similar disease do not significantly accumulate (Figure 2), and only drugs that target dopamine receptors, 5-hydroxytryptamine receptor, adrenergic receptors, and histamine receptors for the treatment of neurological diseases *etc.* cluster relatively closer compared with the others (Figure 2). This phenomenon may be attributed to a variety of targets for the same disease and is significantly evident in anti-cancer drugs. For example, DNA, DNA synthesis-related enzymes, different types of tyrosine kinases, histone deacetylase inhibitors, and proteasome inhibitors, among others, are all anti-cancer targets, which lead to the development of anti-drugs in different clusters. In particular, ARRANON, DACOGEN, ELOXATIN, and VIDAZA target DNA; TARCEVA, TYKEERB, and IRESSA target EGFR (epidermal growth factor receptor); NEXAVAR, SUTENT, GLEEVEC, and SPRYCEL target other tyrosine kinases; ZOLINZA targets histone deacetylases; and VELCADE targets proteasome. Therefore, the aforementioned drugs are not clustered together although all of them are used in treating cancer.

Interestingly, TRISENOX is linked to other tyrosine kinase inhibitors, indicating its tyrosine kinase inhibitory activity. Moreover, no drug that simultaneously inhibits EGFR and other tyrosine kinases has been discovered, hence the need for further studies. Several drugs, such as FUZEON, JANUVIA, and XIFAXAN, among others, are not correlated with any other drugs, indicating that they have no common targets with other drugs or the correlation between these targets and the known ones is not yet clear.

### Product Sales

Some preliminary associations between pharmaceutical targets and sales have been identified in our past research [7]. For example, pharmaceutical sales is positively correlated to the number of drug targets, while the average number of targets of blockbuster drugs seems to be higher than one of common drugs [7]. These results do not, however, indicate the essential correlation between pharmaceutical targets and business value in view of the complex interactive relationship between drugs and targets. A new indicator popularly used in network analysis, betweenness centrality, is further employed in this research. The betweenness centrality of the sampled drug in the drug–drug network and its product sales (Pearson’s correlation coefficient  = 0.371, *P*<0.001) have significant correlation, which further revealing the association between the targets’ bridging effect on drugs and economic value. A drug with high betweenness centrality is often a multi-target drug representing an important mediator in the interaction among different targeted therapeutic drugs. This kind of drugs highly shares and controls certain important targeted conduction pathways for the disease therapies of other drugs and thus has a high probability of becoming a best-selling drug.

## Discussion

In this paper, two networks, namely, the target–target and drug–drug networks (Figure 1 and Figure 2), were visualized using network analysis tools. The drug discovery status and trend were analyzed based on the new NMEs approved by the U.S. FDA from January 2000 to December 2009.

The average target number of sampled drugs from January 2000 to December 2009 is slightly higher than that of the drugs collected by Drugbank before May 2006 [6], [7]. Moreover, the average target number of blockbuster drugs is also higher than that of all our collected samples [7]. These observations indicate that multi-target drug discovery is indeed a status over the past decade and a possible trend in the future, although many single-target drugs are still used today. This development is primarily due to the recent changes in people’s lifestyles, leading to morbidity and alteration in the market share of therapeutic areas. The sales of drugs for nervous and cardiovascular system diseases and anti-neoplastic agents exceed the average sales of all drugs [7]. In fact, cancer and those nervous and cardiovascular system diseases are complicated, thereby promoting the multi-targeted therapies as a better pathway to achieve the desired treatment. For example, the therapeutic targets for cancer include tubulin, topoisomerases, various types of tyrosine kinases, mammalian target of rapamycin, phosphatidylinositol 3-kinase, histone deacetylases, focal adhesion kinase, AMP-activated protein kinase (AMPK), 26S proteasome complex, and cyclooxygenase, among others [8], [9], [10], [11], [12], [13], [14], [15], [16], [17], [18]; the therapeutic targets for Alzheimer’s disease include acetylcholinesterase, secretase, monoamine oxidase B, and τ protein, among others [19], [20], [21], [22], [23]; the therapeutic targets for atherosclerosis include acylcoenzyme A-cholesterol acyltransferase, high density lipoprotein, lectin like oxidized low density lipoprotein receptor, AMPK, and peroxisome proliferator-activated receptor (PPAR), among others [18], [24], [25], [26], [27]. It seems that using single-targeted agents to cure these complex diseases is almost impossible. The multiple tyrosine kinase inhibitor imatinib induces better anti-cancer effects compared with that of gefitinib, which involves a single target [28], further indicating that drugs with multiple targets may exhibit a better chance of affecting the complex equilibrium of whole cellular networks than drugs that act on a single target. Actually, there are several molecular targets, such as dopamine receptors, 5-hydroxytryptamine receptor, adrenergic receptors, cyclooxygenase, monoamine oxidase B, AMPK, PPAR, *etc.* (Fig. 1 and [12], [18], [26], [29], [30], [31]), are common to the complex human diseases, indicts that these targets may play vital roles in the development of complex disease and also suggests that drugs target these targets may have the potential for the secondary development.

Then, how do we develop multi-targeted drugs successfully? Although a number of marketed drugs are thought to derive their therapeutic benefit by interacting with multiple targets, majority of these were discovered accidentally. Therefore, the rational discovery of multi-target drugs is an emerging area. For instance, tyrosine kinases are good targets for the treatment of cancer, and several drugs have already been approved by the U.S. FDA. As targeting several tyrosine kinase receptors at once may dramatically affect the progression of cancer and decrease resistance, some multi-target tyrosine kinase inhibitors have been developed in the recent years [14], [15], [32]. Though there are some studies for multi-target drug design in the recent years [19], [20], [33], it is still a long way to rationally design promising multi-target agents based on current knowledge. The most important thing is that we still not clear which targets should be combined to design better drugs for the specific complex diseases. As natural products are a rich reservoir for drug discovery because of their diversity and complexity structures [34], [35] and most of the natural products are multi-target, we propose that screening the new compounds from natural products based on high content screening is an effective strategy. It is also worthy to re-screening and re-evaluating the dirty compounds such as curcumin [36], [37], [38], [39], [40], berberine [37], [38], [41], and baicalein [42], among others. Of course it is worth noting that there are also several disadvantages of natural products, such as low bioavailability, weak effects, and complex molecular mechanism of actions, among others [34]. Thus, structure modification using medicinal chemistry and pharmaceutical technologies and mechanisms identification using advanced modern technologies are necessary [35].

Combinatorial therapy is another kind of multi-target drug. The treatment of cancer in clinical is almost combination therapy and it is also increasingly used in the prevention and treatment of AIDS, cerebral ischemia, Parkinson’s disease, and Alzheimer’s disease, among others [43], [44], [45], [46]. What will happen if all known targets for one complex disease were simultaneously affected using one compound or drug combination? Identifying such compound or combination is actually impossible, and toxicity is another problem that will be raised. Thus, one better way is to combine the targets selectively according to the developing knowledge and screen the compounds for rational drug discovery. Therefore, the mechanisms causing a particular disease must be clarified. The rapid development of technologies in biological systems such as genomics, proteomics, metabonomics and so on, may enhance our understanding of the nature of the disease, effectively find possible therapeutic targets, and generate computer models that will identify the correct multi-fitting and further make this novel drug design paradigm successful.

In summary, we applied network analysis tools and successfully visualized the information. The approach may still have more or less biases. For example, some targets information may be changed due to the growth of knowledge. Nevertheless, we have confirmed the status of drug discovery in the recent years and put forward the possible future trend.

## Materials and Methods

### Data Sources

All NMEs approved by the U.S. FDA from January 2000 to December 2009 were taken from the Drugs@FDA database. The targets of all sample drugs were individually collected from the Drugbank database in 2011, while the drug–target pairs were constructed accordingly. Furthermore, the therapeutic classification and sales information of the sample drugs were collected from the IMS Health database, a leading pharmaceutical market database in the world, using all NMEs’ brand names as retrieval keywords.

### Network Construction

The drug–target pairs were visualized based on the interaction between the drugs and targets using network analysis tools (Pajek and NetDraw). The original two-mode drug–target network was further constructed, wherein two types of nodes, namely, drugs and targets, and edges represent the strength of interaction between drugs and targets, which is measured by the frequency of their interactions. Thicker ties mean stronger interactions, whereas thinner links represent weaker relationships. Moreover, the two-mode drug–target network was converted into one-mode drug–drug and target–target networks based on the network neighborhood. The drug–drug network only includes drugs as network members, whereas the ties between drugs represent the number of targets simultaneously focused by two neighboring drugs. On the contrary, a target–target network is composed of only target members, their links stand represent the number of drugs that focus on the two neighboring targets.

### Centrality Analysis

Centrality measures the location of network nodes. Betweenness centrality indicates the interval between one node and the other nodes, demonstrating the medium degree of a certain node within the network
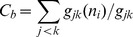
where *g_jk_* denotes the geodesic number between node *j* and node *k* and *g_jk_*(*n_i_*) indicates the geodesic number involving node *n_i_* between two nodes. Thus, the betweenness of node *n_i_* is the sum of *g_jk_*(*n_i_*)*/g_jk_*. The betweenness centrality ranges from 0 to 1∶0 means that the node cannot control any other nodes in the network, whereas 1 indicates that the node seizing the central position in the network can entirely control all other nodes. Herein, the betweenness centrality of nodes in drug and target networks was measured accordingly, and the importance and role of specific drugs and targets in the networks were observed.
